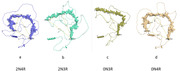# AlphaFold 2 predicts a markedly different MTBR conformation for 3‐repeat and 4‐repeat tau isoforms

**DOI:** 10.1002/alz.089570

**Published:** 2025-01-03

**Authors:** Daniel G Chain, Richard A Margolin, Scott Pollack, Maria Beatriz de Castro V. Goncalves

**Affiliations:** ^1^ TauC3 Biologics Limited, London United Kingdom

## Abstract

**Background:**

Tau abnormalities are a central feature of Alzheimer’s disease (AD) and the defining feature of non‐AD tauopathies, which include frontotemporal lobar degeneration (FTLD) due to Pick’s disease (PiD) or *Mapt* mutations (FTLD‐tau), as well as progressive supranuclear palsy (PSP), corticobasal degeneration (CBD) and others. *Mapt* transcripts undergo alternative splicing to produce 6 distinct isoforms. Exon 2 splicing produces 0, 1 or 2 inserts; exclusion or inclusion of exon 10 results in 3‐repeat (3R) or 4‐repeat (4R) forms, respectively. These forms are differentially present in tauopathies, e.g., 3R in PiD; 4R in almost all FTLD‐tau, PSP, and CBD; and equal 3R/4R in AD. 3R tau is reported to have lower microtubule binding affinity and a higher propensity to form soluble oligomers. Combining structural modeling of tau by AlphaFold 2 (AF2) with biochemical analyses may illuminate the impact of repeat and insert differences. While tau is considered generally disordered, AF2 modeling of 4R tau that we performed predicted a mainly ordered structure for the MTBR from Leu243 to Asp368. Biochemically, we found that the MTBR conformation is altered by some FTD associated mutations and caspase‐3 cleavage. In the present study, we studied the MTBR conformations for the 6 isoforms.

**Method:**

We first analyzed FASTA sequences of the tau isoforms by AF2 and aligned them based on the predicted MTBR conformations. We then compared intramolecular distances between selected MTBR residues.

**Result:**

Absence of the second repeat in 3R tau causes a dramatic difference in the MTBR conformation compared to 4R tau. Specifically, the inward fold N‐terminus to Pro301 is lost, thereby producing an “open” conformation (Figure, b vs a). The number of inserts does not affect the conformation of 4R tau (Figure, d vs. a) but significantly alters that of 3R tau (Figure, c vs. b).

**Conclusion:**

3R tau’s open MTBR conformation may explain its increased propensity to oligomerize and suggests the possibility of additional binding sites that may increase the interactome compared to 4R tau. The absence of the inward fold in 3R tau suggests that this region may be important for microtubule binding.